# Estimating the burden of illness related to genital warts in the Philippines: a nationally representative cross-sectional study

**DOI:** 10.1186/s13027-019-0240-y

**Published:** 2019-10-07

**Authors:** Lani Buenconsejo, Smita Kothari-Talwar, Karen Yee, Amit Kulkarni, Nuria Lara, Montserrat Roset, Anna R. Giuliano, Suzanne Garland

**Affiliations:** 1 Makati Medical Center, Makati, Philippines; 2 Merck & Co. Inc., Kenilworth, NJ USA; 3 Cubist Pharmaceuticals, Lexington, MA USA; 4 IMS Health, Barcelona, Spain; 5 Center for Infection Research in Cancer (CIRC) at Moffitt Cancer Center, Tampa, FL USA; 6 Royal Women’s Hospital, Melbourne, Australia

**Keywords:** Genital warts, Prevalence, Health care cost and utilization, Psychosocial impact

## Abstract

**Background:**

This study estimated genital warts prevalence, genital-warts-related healthcare resource use and costs, and self-reported human-papillomavirus-related psychosocial impact among male and female patients aged 18–60 years in the Philippines.

**Methods:**

Prevalence was estimated using daily logs numbering genital warts patients treated by participating physicians in 4 Philippine regions over a 5-week period (09JUL2011-24SEP2012). Physicians also completed a survey assessing patient referral patterns, healthcare resource use, treatment, and follow-up care. Psychosocial impact was estimated using the human papillomavirus impact profile and the EQ-5D questionnaires. HIP and EQ-5D scores were compared according to the presence of GW (males) and HPV disease (females). CECA scores were also compared by gender and age groups.

**Results:**

Overall genital warts prevalence was estimated at 4.78% (95% confidence interval [CI]: 4.58–4.98%) for men and women aged 18–60 years. Genital warts prevalence was 3.39% (95% CI: 3.13–3.65%) and 8.0% (95% CI: 7.69–8.31%) among women and men, respectively. Prevalence estimates were highest in infectious disease specialist practices 18.67% (95% CI: 18.66–18.69%). Two thirds of the 233 (69.14%) male and 166 (67.20%) female patients were newly-diagnosed genital warts cases. Median costs for genital warts diagnosis and treatment reached 7121 and 7000 Philippine pesos among men and women, respectively. In the *Cuestionario Específico para Condiloma Acuminado* questionnaire, no statistically significant differences between patients were observed. In the EQ-5D questionnaire, male genital warts patients reported lower mean visual analogue scale scores than those without genital warts (78.20 vs 86.34, *p* < 0.0001). Mean visual analogue scale score values and utility values were lower for women with human-papillomavirus-related diseases than those without (77.98 vs 78.93, and 0.84 vs 0.88, respectively).

**Conclusions:**

Genital warts is prevalent in the Philippines; more than 60% of cases were newly diagnosed, contributing to high genital-warts-related healthcare resource costs. Diagnosis of genital warts and human papillomavirus negatively impacted psychosocial indices such as patient well-being and health-related quality of life.

## Background

Human papillomavirus (HPV) causes one of the most common sexually transmitted infections (STIs) [1]. HPV occurs soon after sexual debut and is mostly prevalent in young adults [2]. The more than 130 identified types of this virus are divided into 2 groups according to their epidemiological association with cervical cancer [3]. Low-grade HPV includes subtypes 6 and 11, which are estimated to cause approximately 90% of genital warts (GW) cases [1, 4]. High-risk HPV, including HPV subtypes 16 and 18, induces precancerous lesions such as cervical intraepithelial neoplasia (CIN), cervical cancer, and anogenital cancer [5].

Research suggests that an estimated 6.2 million new GW infections occur annually in individuals aged 14–44 years [6]. However, national incidence data by country is limited, and prevalence estimates by country range widely, from 0.7% in South Korea to up to 25.6% in Nigeria [7–9]. The US National Health and Nutrition Examination Survey found that from 1999 to 2004, 5.6% of survey respondents, aged 18–59 years, self-reported a GW diagnosis [10]. The percentage was higher in women than men: (7.2%; 95% confidence interval [CI]: 6.2–8.4%) vs. (4%; 95% CI: 3.2–5.0%), respectively [10]. A 2015 study using data compiled from regional and national registries from 32 European countries found the estimated annual number of new GW cases ranged between 755,937 and 938,212 in 2015 [11]. In Australia, GW incidence was estimated at 2.19 cases per 1000 with a lifetime prevalence of approximately 4% among Australians aged 16 to 59 years [12–14]. GW incidence has been estimated at 126 per 100,000 person-years in Canada, 203.7 per 100,000 persons-years in Hong Kong, 113.7 per 100,000 person-years in Germany, and 118 per 100,000 person-years in Spain [15]. In Spain, the overall estimated prevalence of GW was 182/100,000 (population) [7].

GW treatment and management weigh heavily on healthcare systems due to the large economic burden associated with repeated physician visits, medication application, and mechanical removal of the warts through cryotherapy [6]. GW treatments can result in significant direct and indirect costs, and with an expected increase in GW incidence, the economic burden is also likely to increase. Two studies evaluating the economic burden of GW in Spain and Germany found similar conclusions related to overall costs. In Germany, overall third party payer (TPP) costs were €49 million and total societal costs were €54.1 million [16]; in Spain, TPP costs were estimated at €47 million and total societal costs at ~€60 million [7]. In Australia, the annual healthcare costs associated with GW management were over A$14 million, with an estimated cost per treated case of ~A$250 and $385 for men and women, respectively [12]. Furthermore, several studies have shown that GW had a negative psychosocial impact on well-being and health related quality of life (HRQL) [17–19]. Patients with GW experienced heavier psychosocial burden than the general population, and females experienced higher burden than males. HRQL dimensions highly impacted for GW depended on the questionnaire used, but those related with self-image and sexual impact were usually highly impacted [19, 20].

To date in the Philippines, limited data exist on the actual epidemiology of GW within the general population due to a lack of research on GW incidence and prevalence. The current data available in the Philippines primarily focuses on cervical cancer where HPV has shown a strong association. In a 1995–98 case study among Filipino cervical cancer cases, the prevalence of all HPV types in the cases was 93.5% [21]. Another study in the Philippines examining the prevalence of HPV infection and cervical abnormalities among 369 female commercial sex workers estimated HPV infection at 57.2% [22]. As such, it is likely that the burden of HPV-related GW is comparatively high in the Philippines, thereby causing a greater economic impact on society and psychosocial burden on individuals.

The aim of this study was to estimate GW prevalence, GW-related healthcare resource use and costs, as well as the self-reported disease-related psychosocial impact among male and female patients aged 18–60 years in the Philippines.

## Methods

### Study design

This was a cross-sectional study conducted to estimate GW prevalence in a 2-week period using physician surveys to approximate healthcare resource use, a daily physician log to assess prevalence, and patient surveys to estimate the psychosocial impact of GW among men and women (18–60 years) in four major cities in the Philippines (Manila, Luzon, Mindanao, and Visayas).

### Prevalence, healthcare costs, and resource use

Referral patterns, healthcare resource use, and costs for GW patients were captured through a 30-min face-to-face physician survey between 09JUL2011-24SEP2012. The survey included questions related to healthcare resource use as part of the usual course of diagnoses, in-office and at-home treatment and procedures, and follow-up care (medical visits, emergency room [ER] visits, hospitalizations). In addition, several survey questions were included to also determine patient referral patterns within the practice, from general practitioners (GPs) to specialists and between specialists.

Secondly, participant physicians completed a daily log during a 2-week period, recording information on the number of patients seen for all causes, including their age and gender; the number of GW patients seen for new or existing disease episodes (whether GW was the primary reason for the visit or was incidentally diagnosed at the time of visit); and the number of patients retained for treatment versus those referred to another specialist for treatment or for follow-up with the general practitioner.

Participating physicians were identified through the Intercontinental Marketing Services (IMS) database in the Philippines, which includes information on approximately 11,344 physicians. For this study, physician locations were selected to reflect all major regions of the country, including the metropolitan area of Manila and key cities in the Luzon, Visayas, and Mindanao regions. The physicians were evenly divided between the public and private sectors.

Physicians were included if they:

- were primary care physicians (PCPs), GPs or family medicine (FM) physicians, obstetrician/gynecologists (OB/GYNs), urologists (UROs), dermatologists (DERMs), or infectious disease specialists (IDSs) with 2–30 years of practice experience;
- devoted ≥50% of their time to treating patients for outpatient visits, ≥3 work days per week;
- (GPs or OB/GYNs) typically handled ≥50 outpatient visits per week;
- (UROs) typically handled ≥30 outpatient visits per week;
- (IDSs or DERMs) typically handled ≥20 outpatient visits per week; and
- treated patients aged 18–60 years for ≥50% of their outpatient visits.

### Psychosocial impact of GW and selected HPV diseases

Physicians who completed the physician survey and the patient daily log were invited to participate in the second phase of the study if they were treating a ≥ 3 patients with GW per month (all specialists) and ≥ 1 patient per month with CIN2/3 (only OB/GYNs). Patients attending to the study investigators office were consecutively invited to participate in a one-time survey and giving them a patient informed consent form with a short description of the study.

Female patients participating in the psychosocial impact analysis were selected based on their new or existing HPV-related outcomes or diagnoses (excluding patients with cervical cancer) within 3 months prior to study recruitment. Women were included if they:

- were aged 18–60 years;
- experienced an HPV-related event within the past 3 months;
- were in good self-reported health; and
- had one of the following:

- ❖ abnormal Papanicolaou (Pap) test results with no definitive histology, conforming to the Bethesda Category-2001 category of squamous or glandular cell abnormality, and no previous high-risk HPV test performed;
- ❖ positive high-risk HPV DNA test results after an abnormal Pap test, as defined above;
- ❖ a diagnosis of external GW or treatment for recurrence;
- ❖ a histological diagnosis of a precancerous or cancerous cervical lesion (eg, CIN1, CIN2, CIN3);
- ❖ (control group) a normal Pap result with no abnormal Pap test or definitive therapy within the past year; or
- ❖ ≥2 of the above conditions (not including GW patients) categorized as upper level disease severity.

Male patients participating in the psychosocial impact analysis had a new or existing external GW case (ie, GW, anal warts, venereal warts) within 3 months prior to study recruitment. Male patients included in the study were aged 18–60 years, were in good self-reported physical health, and belonged to one of the following categories:

- had new or existing external GW within the past 3 months of study recruitment; or
- (control group) did not have a GW diagnosis, GW treatment prescription, or surgery or therapy in the genital area.

Patients were excluded if they were females diagnosed with GW with precancerous cervical lesions, abnormal Pap test results, and HPV-positive test results; had evidence of the presence of any other concurrent/active STI; were concurrently enrolled in clinical studies of investigational agents; had a history of known prior vaccination with HPV vaccine; had recent (≤1 year from enrollment date) or ongoing alcohol or other substance abuse; or were unable to give informed consent.

All data collection was conducted at the workplaces of the participating physicians. To measure psychosocial burden (general health, sexual activity, cervical cancer screening behavior, psychosocial impact, GW experience, socio-demographic information), participants completed the HPV impact profile (HIP), the Cuestionario Específico para Condiloma Acuminado (CECA—specific questionnaire for condylomata acuminata), and the EuroQol-5 Dimension 3 level (EQ-5D) questionnaire, which were translated to Filipino (standardized Tagalog) and culturally pre-tested.

The HIP is a validated 29-item self-administered questionnaire, designed to measure the psychosocial impact of HPV-related health conditions in women [23]. HIP mean scores are categorized as < 40 (no/little impact), 40–70 (moderate impact), and > 70 (high psychological impact). This survey was adapted for use in males in consultation with the original developer and underwent cognitive testing in the United States.

The CECA questionnaire includes 10 questions across 2 domains: emotional and sexual activity [24, 25]. CECA scores range from 0 (worst HRQL) to 100 (best HRQL).

The EQ-5D is a 2-part questionnaire, including descriptive and thermometer or Visual Analogue Scale (VAS), and serves as a generic validated instrument for use as a measure of HRQL [26]. VAS scores range from 0 (death) to 100 (perfect health).

### Statistical analysis

#### Prevalence, health care costs, and resource use

The estimate for GW prevalence in physician practices was calculated using the number of new or existing GW cases observed, divided by the total number of visiting patients during the 2-week study period. Prevalence was calculated for overall patients, and for each physician specialty type, age group, and sex, reporting also the 95% CI. Previously-diagnosed GW patients who sought medical care for other reasons were not considered. A national-level prevalence estimate was extrapolated from the estimated prevalence for each specialty and the distribution of GW patients seeking healthcare among the specialties. The national-level proportion of GW patients who sought care was calculated according to physician specialty, based on the following formula:

$$ Gi=\left( Si\ast Di\right)/\left(\varSigma i= 1\  to\  4\  Si\ast Di\right), $$

where *S* is the number of physicians of a given specialty in the country, *D* is the mean number of GW patients seen by the specific specialty (based on 2-week daily logs), and *i* is the specialty type (PCP, GP/FM, OB/GYN, URO, DERM, IDS). The proportions of patients seeing each specialty type were used in the following formula to derive weights applied to patients included in the study database, and to derive a national-level prevalence estimate:

$$ \mathrm{W}=\left(\mathrm{Gi}\ast \mathrm{nT}\right)/\left(\mathrm{ni}\right), $$

where Gi is the proportion of GW patients at a national level treated within each specialty, *nT* is the total number of patients included in the study, and *ni* is total number of patients counted in the study for a given specialty.

Referral patterns reported by physicians were described, reporting the percentage of patients consulted directly by PCP, URO, or DERM as well as those referred by another physician. Healthcare resource use reported by participant physicians in the corresponding survey was described and compared between the physician specialty types. Costs reported in Philippine pesos (PHP) were calculated based on healthcare resource use reported by physicians in the corresponding survey and reported by physician specialty.

#### Psychosocial impact of GW and selected HPV diseases

Using the HIP score, survey items were linearly transformed to a 0–100 scale, with higher scores indicating better conditions. To account for missing data, scale scores were created by computing the mean as the sum of the item scores over the number of items answered. If more than 50% of the items on the scale were missing, the score was not computed. To create the total scale score, the mean was computed as the sum of all the items over the number of items answered on all scales.

The Japanese version of the EQ-5D instrument was used in this study to estimate the utilities associated with EQ-5D health status [27]. Japan was the first Asian country to develop its own preference weights of EQ-5D since 2002, and the model was chosen to represent Asian preference weights [28]. VAS scores range from 0 (worst HRQL) to 100 (best HRQL), and utility values from 0 (death) to 1 (perfect health).

HIP and EQ-5D scores were compared according to the presence of GW diagnosis (for males) and HPV disease diagnosis (for females). For continuous variables, the Student T-test or Mann-Whitney U-test was performed to compare normal and non-normal variables between subgroups. For categorical variables, Chi-square or Fisher’s exact tests were performed depending on the distribution of patients across response categories. CECA scores were also compared, just for patients with GW, by gender and age groups (18–29 years, 30–44 years, and 45–60 years) using the Mann-Whitney U-test and Kruskal-Wallis test, respectively.

## Results

### Prevalence

A total of 157 physicians (28 PCPs, 43 OB/GYNs, 29 UROs, 29 DERMs, 28 IDSs) participated in the study. The overall prevalence was estimated at 4.78%, with a higher overall prevalence in men (8.00%) compared with women (3.39% (Table 1), data adjusted by specialty). However, overall prevalence decreased to 2.66% (95% CI: 2.43–2.89) once adjusted by physician specialty, age, and gender according to the distribution of general population in Philippines.

**Table 1:** GW Prevalence in the Philippines by Patient Sex and Physician Specialty

	Male		Female		Overall	
	(n/N)	(%, 95 CI)^2^	(n/N)	(%, 95 CI)^2^	(n/N)	(%, 95 CI)^2^
PCP	7/1590	0.44 (0.44;0.44)	4/1745	0.23 (0.23;0.23)	11/3335	0.33 (0.33;0.33)
OB/GYN	1/39	2.56 (2.51;2.61)	58/2724	2.13 (2.12;2.14)	59/2763	2.14 (2.13; 2.14)
URO	41/1009	4.06 (4.05;4.08)	0/169	0	41/1178	3.48 (3.47;3.49)
DERM	21/554	3.79 (3.78;3.81)	11/987	1.11 (1.11:1.12)	32/1541	2.08 (2.07;2.08)
IDS	267/1358	19.7 (19.64;19.68)	174/1004	17.3 (17.3;17.4)	441/2365	18.7 (18.7;18.7)
OVERALL^1^	337/4550	8.0 (7.69;8.31)	247/6629	3.39 (3.13;3.65)	584/11179	4.78 (4.58;4.98)

^1^Data weighted according to specialty type

^2^Percentage and 95% CI calculated considering the number of patients with identified GW status

Data weighted according to specialty type

*CI* confidence interval; *DERM* dermatologist; *GW* genital warts; *IDS* infectious disease specialist; *OB/GYN* obstetrician/ gynecologist; *PCP* mprimary care physician, *URO* urologists

**Fig. 1:**
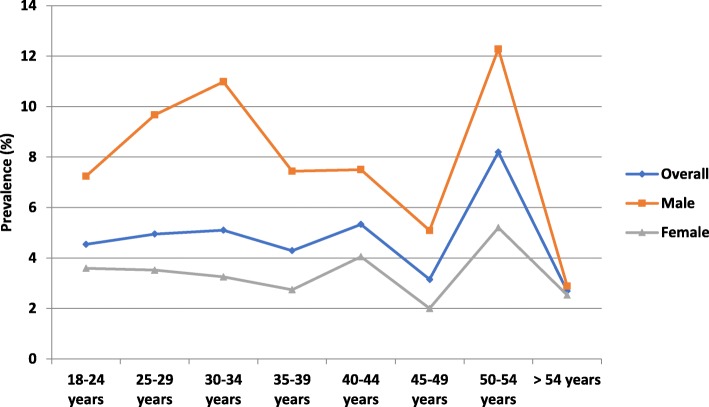
GW Prevalence by Age and Sex (Weighted). GW = genital warts

Regarding GW prevalence rates by patient age, patients aged 50–54 years had the highest rate (8.20%), while those aged > 54 had the lowest (2.69%) (Fig. 1). Prevalence by gender and physician specialty showed that patients treated by an IDS had a high prevalence among male (19.7%) and female (17.3%) patients (Table 1). In addition, prevalence was higher for male patients treated by URO (4.06%) (Table 1).

For GW patients with physician office visits during the 2-week study period, the percentage of patients with existing GW instead of new GW were higher for OB/GYN practices (40.68%) followed by PCP (36.36%), and IDS (33.78%); and being lower in other practices like DERM (18.75%) or URO (4.87%). The distribution of existing cases between recurrent and resistant cases also varied by specialty. The percentage of resistant GW cases ranged from 0% for PCP and URO consultations to 38.88% for IDS consultations (Table 2).

**Table 2:** Description of GW Cases by Specialty

	PCP (*n* = 28)	DERM (*n* = 29)	OB/GYN (*n* = 43)	URO (*n* = 29)	IDS (*n* = 28)
All GW Patients	11 (0.30%)	32 (2.10%)	59 (2.10%)	41 (3.50%)	441 (18.50%)
New or existing GW					
New Case	7 (63.64%)	26 (81.25%)	35 (59.32%)	39 (95.13%)	292 (66.22%)
Existing Case	4 (36.36%)	6 (18.75%)	24 (40.68%)	2 (4.87%)	149 (33.78%)
Valid n	11	32	59	41	441
Existing cases					
Recurrent	4 (100.00%)	5 (83.33%)	20 (83.33%)	2 (100.00%)	91 (61.12%)
Resistant	0 (0.00%)	1 (16.67%)	4 (16.67%)	0 (0.00%)	58 (38.88%)
Valid n	4	6	24	2	149

^1^ **New Case:** GW case **not** diagnosed previously by patient or another physician

^2^ **Existing Case:** GW case **was** diagnosed previously by patient or another physician

^3^ **Recurrent Case:** GW case where previous episodes **were** resolved with treatment

^4^ **Resistant Case:** GW case where previous episodes **were not** resolved with treatment

*DERM* dermatologist, *GW* genital warts, *IDS* infectious disease specialist, *OB/GYN* obstetrician/gynecologist, *PCP* primary care physician, *URO* urologists

### Referral patterns, healthcare resource use and costs

**Fig. 2:**
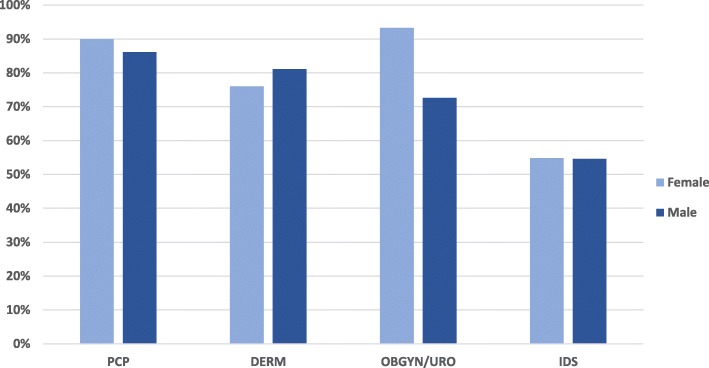
GW Patients Initially Treated by Consulting Physicians. DERM = dermatologist; GW = genital warts; IDS = infectious disease specialist; OBGYN = obstetrician/gynecologist; PCP = primary care physician; URO = urologist

The percentages of male and female patients who consulted directly with that physician without referral were 86.1 and 90.0% for PCPs, 81.07 and 76.0% for DERMs, 72.6% for UROs (males), 93.26% for OBSGYN (females), and 54.64 and 54.8% for IDSs (Fig. 2). Following GW diagnosis, the percentages of patients by gender treated by the different physician specialties were 48.5% (males) and 30.0% (females) for PCP 76.9% (males) and 77.4% (females) for DERM, 98.3% (males) for URO, 97.3% (females) for OBGYN, and 81.6% (males) and 63.0% (females) for IDS. The mean (SD) number of office visits performed during a GW episode (from initial diagnosis to close of treatment), as reported by participating physicians, was 3.74 (2.29) for males and 3.50 (2.33) for females, both with a range of 1 to 20 visits per episode (minimum and maximum, respectively). The mean number of visits reported was similar across physician specialties, ranging in males from 3.48 for DERM to 4.25 for IDS and in females from 3.00 for PCP to 4.08 for IDS. The number of hospital or ER visits was approximately one-tenth of the number of office visits for the treatment of 1 GW episode. Physicians reported a mean (SD) of 0.21 (1.02) hospital or ER visits per episode in males and 0.3 (0.99) hospital or ER visits per episode in females (Table 3).

**Table 3:** Health Care Utilizations for GW Patients

	PCP (*n* = 28)	DERM (*n* = 29)	URO (*n* = 29)	IDS (*n* = 28)	Overall (*n* = 114)
Male Patients					
Number of office visits					
Mean	3.50	3.48	3.59	4.25	3.74
SD	1.51	1.53	1.66	3.47	2.29
Median	3.5	3.0	3.0	3.0	3.0
Min	1.0	2.0	1.0	2.0	1.0
Max	6.0	7.0	8.0	20.0	20.0
Valid n	12	27	29	28	96
Number of hospital or ER visits					
Mean	0.00	0.72	0.08	0.00	0.21
SD	0.00	1.88	0.41	0.00	1.02
Median	0.0	0.0	0.0	0.0	0.0
Min	0.0	0.0	0.0	0.0	0.0
Max	0.0	8.0	2.0	0.0	8.0
Valid n	19	25	24	27	95
Female Patients					
	PCP (*n* = 28)	DERM (*n* = 29)	OB/GYN (*n* = 43)	IDS (*n* = 29)	Overall (*n* = 129)
Number of office visits					
Mean	3.00	3.83	3.16	4.08	3.50
SD	1.71	2.60	1.17	3.57	2.33
Median	2.0	3.0	3.0	3.0	3.0
Min	1.0	0.0	1.0	2.0	0.0
Max	6.0	12.0	6.0	20.0	20.0
Valid n	14	24	43	24	105
Number of hospital or ER visits					
Mean	0.00	0.65	0.40	0.07	0.30
SD	0.00	1.58	1.04	0.38	0.99
Median	0.0	0.0	0.0	0.0	0.0
Min	0.0	0.0	0.0	0.0	0.0
Max	0.0	6.0	4.0	2.0	6.0
Valid n	22	23	42	27	114

*DERM* dermatologist, *ER* emergency room, *GW* genital warts, *IDS* infectious disease specialist, *min* minimum, *max* maximum, *OB/GYN* obstetrician/gynecologist, *PCP* primary care physician, *SD* standard deviation, *URO* urologists

**Fig. 3:**
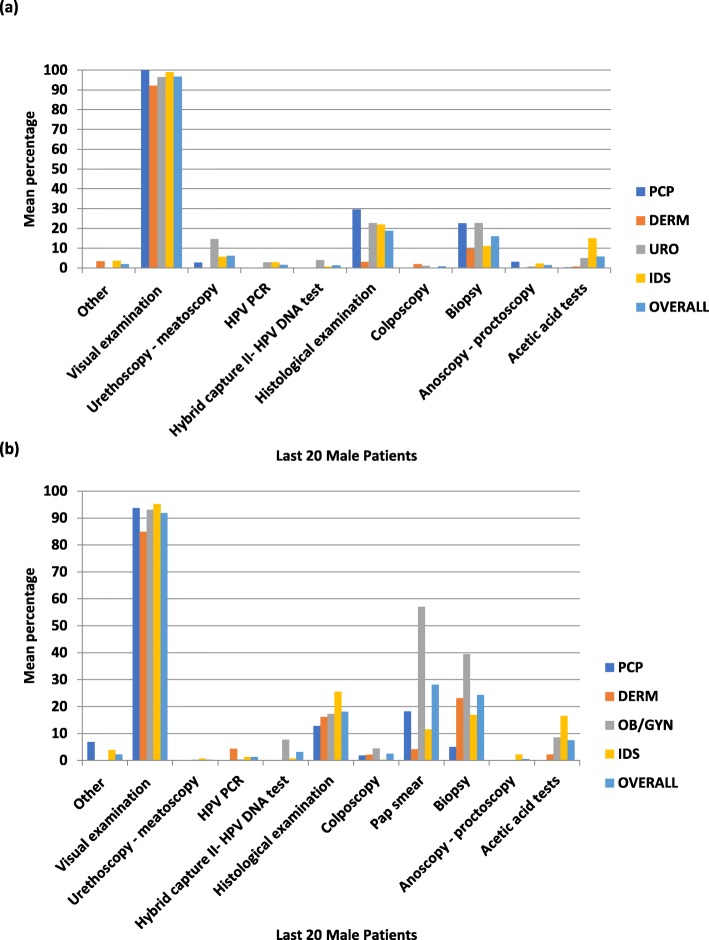
**a** GW Diagnostic Tool and Technique Utilization for GW Treatment for Male Patients. DERM = dermatologist; DNA = deoxyribonucleic acid; GW = genital warts; HPV = human papillomavirus; IDS = infectious disease specialist; PCP = primary care physician; PCR = polymerase chain reaction; URO = urologist. **b** GW Diagnostic Tools and Techniques Distribution for Female GW Patients . DERM = dermatologist; DNA = deoxyribonucleic acid; GW = genital warts; HPV = human papillomavirus; IDS = infectious disease specialist; Pap = Papanicolaou test; PCP = primary care physician; PCR = polymerase chain reaction; OB/GYN = obstetrician/ gynecologist

Visual examination was the primary diagnostic technique used by all specialists (Fig. 3). In males, except for biopsy and histological examination, all other tools and techniques (eg, colposcopy, anoscopy-proctoscopy, hybrid capture II-HPV DNA tests) were seldom used to diagnose GW. In females, OB/GYNs used most test types, including the Pap smear, more frequently than other specialty physicians. IDS physicians administered visual and histological examinations as well as acetic acid tests more frequently.

**Fig. 4:**
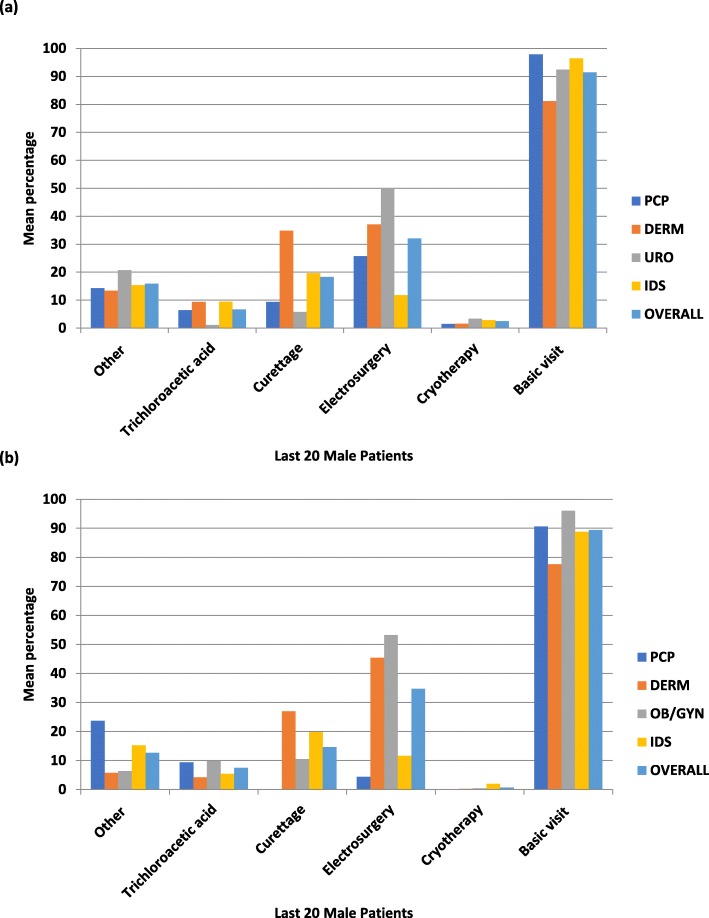
**a** In-office Treatment/Procedures for Male GW Patients. DERM = dermatologist; GW = genital warts; IDS = infectious disease specialist; PCP = primary care physician; URO = urologist. **b** In-office Treatment/Procedures for Female GW Patients. DERM = dermatologist; GW = genital warts; IDS = infectious disease specialist; OB/GYN = obstetrician/ gynecologist; PCP = primary care physician

**Fig. 5:**
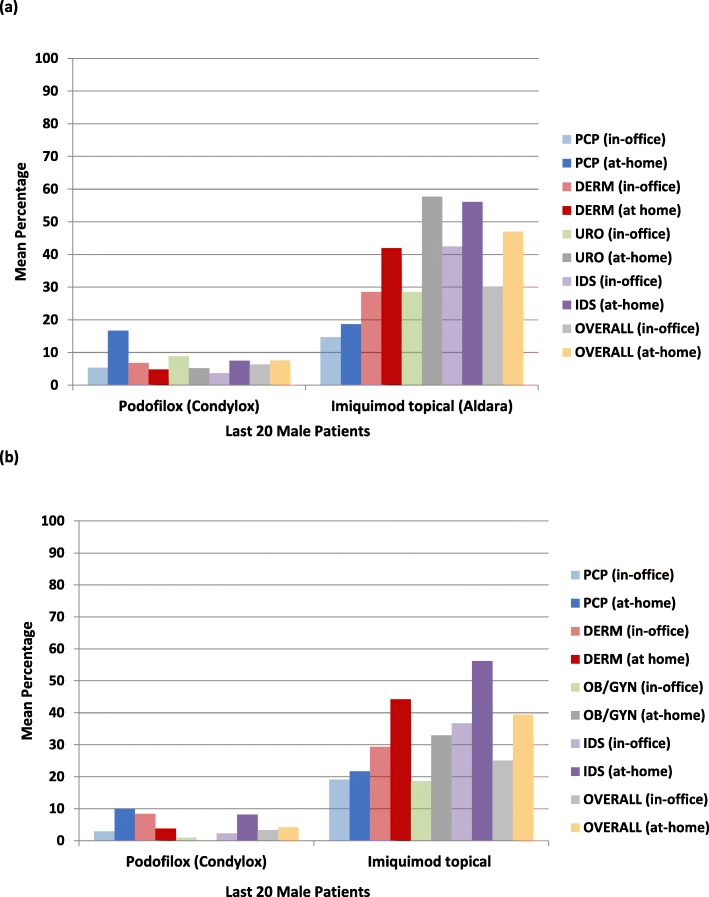
**a** Male GW Patients Prescribed In-office and At-home Topical Medication. DERM = dermatologist; GW = genital warts; IDS = infectious disease specialist; PCP = primary care physician; URO = urologist. **b** Female GW Patients Prescribed In-office and At-home Topical Medication. DERM = dermatologist; GW = genital warts; IDS = infectious disease specialist; OB/GYN = obstetrician/ gynecologist; PCP = primary care physician

In terms of treatments used, electrosurgery was more frequently used by URO, OB/GYN, and DERM physicians; and curettage was utilized more often by DERMs (Fig. 4). In addition to topical medications administered in-office, ~ 50% of GW patients were also prescribed topical medications for at-home use. Imiquimod topical (Aldara®) was more frequently administered by IDSs and DERMs, and it was prescribed more frequently as an at-home rather than an in-office medication by all specialists (Fig. 5a).

**Fig. 6:**
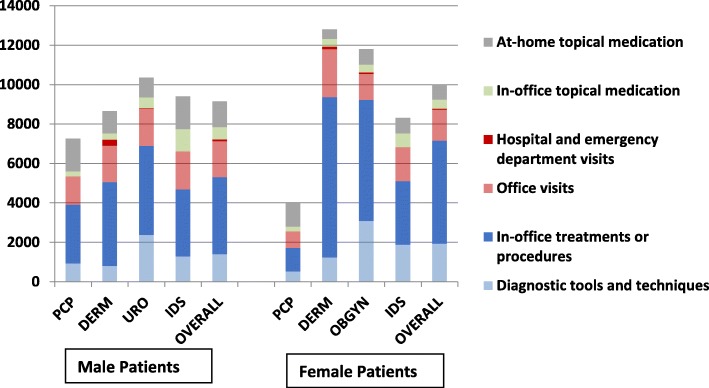
Mean Costs Associated with GW Diagnosis and Treatment (Philippine Pesos). DERM = dermatologist; GW = genital warts; IDS = infectious disease specialist; PCP = primary care physician; OB/GYN = obstetrician/gynecologist; URO = urologist

Figure 6 shows the mean costs associated with GW diagnosis and management among male and female patients, overall and by specialty. Female patients incurred higher GW-related costs, as reported by DERMs, followed by OB/GYNs. In male patients, higher costs were reported by UROs, followed by IDS specialists. Healthcare resource utilizations, the overall median and mean costs associated with a GW episode (diagnosis and treatment), were estimated at 7121.30 PHP and 8105.52 PHP (SD: 5823.79), respectively, for men, and 7000.00 PHP and 8786.93 PHP (SD: 9576.08), respectively, for women.

### Psychosocial impact of GW and selected HPV diseases

HIP showed statistically significant differences among male patients with and without GW for all scores, except control/life impact (*p* = 0.187). In all statistically significant scores, males with GW patients reported higher values, reflecting a higher disease impact (scale: 0–100) compared with patients without GW (Table 4). Among female patients, no statistically significant differences were found among those diagnosed with selected HPV diseases in any of the HIP questionnaire domains (Table 4). However, comparisons between study groups showed statistically significant differences between patients with external GW and those with abnormal Pap and HPV positive test results, showing higher disease impact in patients with GW. These differences were identified in the following dimensions: “worries and concerns” (*p* = 0.018), “emotional impact” (*p* < 0.001), “partner issues and transmissions” (*p* = 0.007), and “interaction with doctors” (*p* < 0.001) (Table 8 in Appendix). Differences were not statistically significant among HPV-related disease groups regarding sexual impact, self-image, and control-life impact.

**Table 4:** HIP Questionnaire Scores per GW Diagnosis

Male Patients (*n* = 149)	With GW (*n* = 74)	No GW (*n* = 75)	Overall	*P*-value
HPV Impact Profile Total Score				
Mean	52.35	37.72	44.99	< 0.0001
SD	11.81	17.04	16.37	
95% CI	(49.6; 55.1)	(33.8; 41.7)	(42.3; 47.7)	
Worries and Concerns				
Mean	55.85	31.38	43.62	< 0.0001
SD	20.70	25.20	26.06	
95% CI	(51.0; 60.7)	(25.5; 37.3)	(39.4; 47.9)	
Emotional Impact				
Mean	48.06	31.55	39.75	< 0.0001
SD	16.22	20.56	20.25	
95% CI	(44.3; 51.8)	(26.8; 36.3)	(36.4; 43.0)	
Sexual Impact				
Mean	49.45	43.11	46.26	0.0199
SD	14.71	17.80	16.59	
95% CI	(46.0; 52.9)	(39.0; 47.2)	(43.6; 49.0)	
Self-image				
Mean	46.27	40.74	43.49	0.0371
SD	14.80	16.94	16.10	
95% CI	(42.8; 49.7)	(36.8; 44.7)	(40.9; 46.1)	
Partner Issues and Transmission				
Mean	60.89	48.09	54.63	0.0008
SD	15.97	26.90	22.84	
95% CI	(57.1; 64.7)	(41.6; 54.6)	(50.8; 58.5)	
Interactions with Doctors				
Mean	55.38	35.85	46.61	< 0.0001
SD	17.08	22.41	21.87	
95% CI	(51.3; 59.5)	(29.9; 41.8)	(42.8; 50.5)	
Control/Life Impact				
Mean	50.41	46.49	48.44	0.1868
SD	13.98	21.12	17.98	
95% CI	(47.1; 53.7)	(41.6; 51.4)	(45.5; 51.4)	
Female Patients (*n* = 225)	HPV disease (*n* = 182)	No HPV disease (*n* = 43)	Overall	*P*-value
HPV Impact Profile Total Score				
Mean	47.98	48.06	47.99	0.9800
SD	19.85	20.56	19.94	
95% CI	(45.1; 50.9)	(41.7; 54.5)	(45.4; 50.6)	
Worries and Concerns				
Mean	49.56	49.11	49.47	0.9252
SD	27.11	30.74	27.75	
95% CI	(45.6; 53.5)	(39.5; 58.7)	(45.8; 53.1)	
Emotional Impact				
Mean	46.72	45.62	46.52	0.7943
SD	24.68	24.71	24.64	
95% CI	(43.1; 50.3)	(37.9; 53.3)	(43.3; 49.8)	
Sexual Impact				
Mean	49.02	51.58	49.47	0.5165
SD	22.51	19.77	22.03	
95% CI	(45.7; 52.3)	(45.1; 58.1)	(46.5; 52.4)	
Self-image				
Mean	43.59	44.31	43.72	0.8314
SD	19.37	21.09	19.66	
95% CI	(40.7; 46.4)	(37.7; 50.9)	(41.1; 46.3)	
Partner Issues and Transmission				
Mean	55.28	54.91	55.22	0.9375
SD	26.02	27.37	26.19	
95% CI	(51.4; 59.2)	(45.6; 64.2)	(51.7; 58.8)	
Interactions with Doctors				
Mean	42.72	41.30	42.46	0.7265
SD	23.76	22.05	23.41	
95% CI	(39.2; 46.2)	(34.3; 48.3)	(39.4; 45.6)	
Control/Life Impact				
Mean	49.83	54.88	50.77	0.1134
SD	18.94	16.88	18.64	
95% CI	(47.1; 52.6)	(49.6; 60.1)	(48.3; 53.2)	
HPV Impact Profile Total Score Categorized				
No or little impact	64 (35.2%)	15 (34.9%)	79 (35.1%)	0.9983
Moderate impact	88 (48.4%)	21 (48.8%)	109 (48.4%)	
Heavy psychological impact	30 (16.5%)	7 (16.3%)	37 (16.4%)	
Valid n	182	43	225	

*HPV* human papillomavirus, *HIP=HPV* impact profile, *CI* confidence interval, *GW* genital warts, *SD* standard deviation

No significant differences were observed in CECA values for each dimension by gender (CECA mean score; men: 4.19 vs women: 4.10; *p* = 0.844) (Table 5) and age groups (CECA mean score; 18–29 years: 3.91, 30–44 years: 4.12, and 45–60 years: 4.17; *p* = 0.710).

**Table 5:** CECA Questionnaire Scores from GW Patients

Patients with GW (*n* = 121)				
	Male (*n* = 74)	Female (*n* = 47)	Overall	*P*-value
Emotional health				
Mean	6.51	6.63	6.56	0.8692
SD	3.75	4.65	4.11	
95% CI	(5.6; 7.4)	(5.3; 8.0)	(5.8; 7.3)	
Sexual activity				
Mean	11.54	10.64	11.18	0.4637
SD	6.16	7.08	6.52	
95% CI	(10.1; 13.0)	(8.6; 12.7)	(10.0; 12.4)	
CECA total score				
Mean	4.19	4.10	4.15	0.8437
SD	2.13	2.60	2.32	
95% CI	(3.7; 4.7)	(3.3; 4.9)	(3.7; 4.6)	

*CECA* Cuestionario Específico para Condiloma Acuminado (Specific questionnaire for Condylomata Acuminata), *CI* confidence interval, *GW* genital warts, *SD* standard deviation

Male patients with GW reported significantly lower mean VAS scores in the EQ-5D (78.2) than those without GW (86.3, *p* < 0.001) (Table 6); results that reflect the impact of a GW diagnosis on male patients. Utility values were also significantly lower for male GW patients (mean: 0.81) compared with male patients without GW (mean: 0.94, *p* < 0.001). In female patients, mean VAS scores (78.0) and utility values (0.84) in patients with selected HPV-related diseases were lower than scores for those without selected HPV-related diseases (78.9 and 0.88, respectively), but the differences were not statistically significant. Like with HIP scores, comparisons between study groups in females showed worse HRQL for patients with GW than those with abnormal Pap and HPV positive test results (VAS: mean scores of 72.9 vs 80.8, respectively; utility values: mean scores of 0.79 vs 0.89, respectively). Differences were statistically significant only for utility values (*p* = 0.005), not for VAS (0.087).

**Table 6:** EQ-5D, VAS Score and Utility: Patients with and without GW

Male Patients (*n* = 149)	With GW (*n* = 74)	No GW (*n* = 75)	Overall	*P*-value
Mobility				
I have no problems in walking about.	64 (88.9%)	67 (93.1%)	131 (91.0%)	0.3830
I have some problems in walking about.	8 (11.1%)	5 (6.9%)	13 (9.0%)	
Self-Care				
I have no problems with self-care.	66 (90.4%)	71 (98.6%)	137 (94.5%)	0.0306
I have some problems washing or dressing myself.	7 (9.6%)	1 (1.4%)	8 (5.5%)	
Usual Activities				
I have no problems with performing my usual activities.	62 (84.9%)	69 (95.8%)	131 (90.3%)	0.0263
I have some problems with performing my usual activities.	11 (15.1%)	3 (4.2%)	14 (9.7%)	
Pain-Discomfort				
I have no pain or discomfort.	43 (58.9%)	60 (85.7%)	103 (72.0%)	0.0015
I have moderate pain or discomfort.	29 (39.7%)	10 (14.3%)	39 (27.3%)	
I have extreme pain or discomfort.	1 (1.4%)		1 (0.7%)	
Anxiety-Depression				
I am not anxious or depressed.	30 (41.7%)	63 (90.0%)	93 (65.5%)	< 0.0001
I am moderately anxious or depressed.	40 (55.6%)	6 (8.6%)	46 (32.4%)	
I am extremely anxious or depressed.	2 (2.8%)	1 (1.4%)	3 (2.1%)	
VAS (EQ-5D)				
Mean	78.20	86.34	82.33	< 0.0001
SD	13.63	10.62	12.82	
95% CI	(75.0; 81.4)	(83.9; 88.8)	(80.2; 84.4)	
Utility Values				
Mean	0.81	0.94	0.87	< 0.0001
SD	0.16	0.12	0.15	
95% CI	(0.8; 0.8)	(0.9; 1.0)	(0.8; 0.9)	
Female Patients (*n* = 225)	HPV (*n* = 182)	No HPV (*n* = 43)	Overall	*P*-value
Mobility				
I have no problems in walking about.	163 (90.1%)	39 (90.7%)	202 (90.2%)	0.8987
I have some problems in walking about.	18 (9.9%)	4 (9.3%)	22 (9.8%)	
Self-Care				
I have no problems with self-care.	172 (96.6%)	42 (97.7%)	214 (96.8%)	0.7254
I have some problems washing or dressing myself.	6 (3.4%)	1 (2.3%)	7 (3.2%)	
Usual Activities				
I have no problems with performing my usual activities.	158 (87.3%)	39 (90.7%)	197 (87.9%)	0.7684
I have some problems with performing my usual activities.	22 (12.2%)	4 (9.3%)	26 (11.6%)	
I am unable to perform my usual activities.	1 (0.6%)		1 (0.4%)	
Pain-Discomfort				
I have no pain or discomfort.	124 (68.1%)	35 (81.4%)	159 (70.7%)	0.2178
I have moderate pain or discomfort.	57 (31.3%)	8 (18.6%)	65 (28.9%)	
I have extreme pain or discomfort.	1 (0.5%)		1 (0.4%)	
Anxiety-Depression				
I am not anxious or depressed.	88 (48.6%)	26 (60.5%)	114 (50.9%)	0.3770
I am moderately anxious or depressed.	82 (45.3%)	15 (34.9%)	97 (43.3%)	
I am extremely anxious or depressed.	11 (6.1%)	2 (4.7%)	13 (5.8%)	
VAS (EQ-5D)				
Mean	77.98	78.93	78.17	0.7121
SD	14.89	15.91	15.06	
95% CI	(75.8; 80.2)	(74.0; 83.8)	(76.2; 80.2)	
Utility Values				
Mean	0.84	0.88	0.84	0.1010
SD	0.15	0.14	0.15	
95% CI	(0.8; 0.9)	(0.8; 0.9)	(0.8; 0.9)	

*CI* confidence interval, *EQ-5D* EuroQol-5 dimension, *GW* genital warts, *HPV* human papillomavirus, *SD* standard deviation, *VAS* visual analogue scale

## Discussion

This cross-sectional study estimated the burden of GW in the Philippines by obtaining the GW prevalence rate, GW-related resource utilization and costs, and data on the self-reported HPV disease-related psychosocial impact among male and female patients aged 18–60 years.

At the national level, the current study estimated GW prevalence at 8.00% among men and 3.39% among women, which is higher than figures reported in currently available literature [7, 29, 30]. However, overall prevalence decreased to 2.66% once adjusted by age, gender, and physician specialty. A previous study performed in Hong Kong, including 170 private doctors working in Social Hygiene Clinics and using the same study approach, estimated an overall GW prevalence rate of 0.94% [15]—significantly lower than this study’s findings on the Philippines.

Recent studies in Europe, the United States, and Australia are focused primarily on estimating GW incidence rather than prevalence. However, the results of a 2007 study of women aged 18–45 years in 4 northern European countries over a 12-month period showed a GW prevalence of 1.3% in Denmark, 1.9% in Iceland, 1.1% in Norway, and 1.0% in Sweden [31]. A recent study performed in Peru using the same methodology reported also similar prevalence rates [32].

The difference in the prevalence of GW between the European studies and this study on the Philippines may be related to sexual behavior differences or differing methods in collecting GW patient data. In the European studies, the median number of sexual partners during a patient’s lifetime was 5, and the median age at the time of first intercourse was 16 years [31], compared to medians of 2 sexual partners and an age of 20 years in the Philippines. However, the median number of sexual partners in the Philippines study accounted only for the most recent 5 years, not a patient’s entire lifetime, as reported in the European study. Differences in terms of age at first diagnosis may also be related to the methods used to collect patient data [33].

Considering age distribution, GW diagnoses differed by gender. In this study, the oldest (aged 50–54 years) female patients showed the highest prevalence. Since most HPV infections occurred soon after sexual debut and are transient, women who are over age 30 and HPV positive include persistent carriers and those with new infections. While most studies show a decrease in HPV prevalence with age, many studies conducted in several different international regions have indicated a peak prevalence of HPV infection in women below age 25 [12], a decrease among women aged 35–54, and a second HPV prevalence peak after age 55 [34].

The study found that GW prevalence was higher in the Philippines among men than for women for all age groups. Nevertheless, a notably higher prevalence was recorded for all patients aged 50–54 years. The prevalence tendency exhibited by male patients according to age, excluding those aged 50–54 years, was similar to the prevalence reported in a Korean study examining the epidemiological characteristics of genital herpes and condyloma acuminata among patients presenting to URO and OB/GYN clinics. Analysis by age group and physician specialty showed a high prevalence among male patients treated by an IDS. It is possible these patients have self-selected their specialist; the referral pattern may show a selection of those thought to have the better knowledge of GW management. The predominant age group of patients with condyloma acuminata was 25–29 years in Korean male patients, with a prevalence of 1.84% [35]. A separate retrospective study of male patients attending a sexually transmitted infection clinic in India reported that 59.7% of sexually transmitted disease cases occurred among patients aged 25–44 years [29].

Most GW patients included in this study were newly diagnosed cases (66.06%). Among those with existing GW, 67.48% were recurrent and 35.52% were resistant. There is currently no previously published data related to the proportion of new or existing GW cases in the Philippines. However, compared with a previous study using similar methodology but performed in Hong Kong [15], a different distribution was observed; approximately one-tenth (73) of the 721 episodes were designated as new cases. The differences in results may suggest that patients in the Philippines have access to better treatment, and therefore cases are less resistant and recurrent. However, it is possible that lesions presented may have not been noted by the physician, leading to a lower proportion of patients with existing GW (33.94%) being reported compared to that observed in Hong Kong. Results obtained in terms of healthcare resource use are aligned with other countries in which the objectives of the current study have been also assessed using the same methodology [36, 37].

Diagnosis and treatment of GW was associated with a median cost of 7121 PHP (~$134 USD) for men, and 7000 PHP (~$132 USD) for women, which is about 8.58% of the average GDP per capita in the Philippines from 1960 to 2016 [38]. It also equals about 3.63% of 2012 annual family expenditures in the Philippines [39]. In-office treatment and procedures (50.46% of the total) were the highest overall cost drivers for male participants, followed by office visits (24.74%), and diagnostic tools and techniques (12.60%). For female participants, cost drivers were in-office treatments and procedures (53.38%), followed by office visits (20.02%), and diagnostic tools and techniques (15.85%). Costs associated with GW have been assessed in several other countries, including the United States, Canada, and Australia, but the socioeconomic situations and healthcare systems in these countries are not comparable to those in the Philippines.

When analyzing the psychosocial impact of GW on men, those with GW reported worse HRQL scores than those without. Despite the significant differences (particularly among men) in the results of the global HIP scores and EQ-5D questionnaires, scores were similar for those with and without the disease, reflecting a lack of psychological impact in these areas. In the disease-specific HIP questionnaire, GW patients reported worse HRQL scores compared to those without GW. On HRQL, female GW patients reported a higher psychosocial impact of the disease than male GW patients. The results align with differences reported in previous studies in other disease areas [40–42].

When comparing female HPV-related disease subgroups, results showed a higher psychological impact on female GW patients than on those with other HPV diseases, including precancerous lesions and CIN2/3. In the HIP questionnaire, GW patients reported experiencing a major impact, especially related to “worries and concerns,” “emotional impact,” “partner issues and transmissions,” and “interactions with doctors.” The higher disease burden on patients may be associated with the presence of physical lesions visual to the patient, similar to a previous study that examined GW burden [12]. In a recently published study conducted in India including HIV, HPV, and herpes patients, World Health Organization Quality of Life-BREF scores for patients in different STI groups were significantly lower than for those without these conditions [43]. In South Korea, a recent study analyzed questions posted by patients on an HPV informational website [44] and concluded that, based on the type of questions posted, GW can be considered an embarrassing topic for patients.

The current study detected a lower impact on patients with GW (assessed by EQ-5D) than previous studies reported. According to Sénécal et al [45]. in a study in Canada, GW was associated with lower HRQL scores for “pain-discomfort,” “anxiety-depression,” and “usual activities.” The same Canadian study also showed an absolute difference in EQ-5D utility scores and EQ-VAS health status between GW patients and the population norms of 9.9 (95% CI: 7.3 to 12.5) and 6.0 (95% CI: 4.1 to 7.9) percentage points, respectively. In the current study, the differences in mean VAS and EQ-5D utility scores between patients with and without GW (and other selected HPV-related diseases in female patients) were 8.1 and 0.1 in men, and 0.95 and 0.04 in women, respectively.

The study results are consistent with previously reported results by Wang et al., in China [46]. According to this study, female GW patients had the highest mean HIP scores (52.2), showing a high psychological impact, followed by:

- patients with precancerous cervical lesions (48.6);
- HPV-positive after abnormal Pap (45.8);
- abnormal Pap test results without an HPV test (44.1);
- HPV diagnosis after abnormal Pap results (43.1); and
- normal Pap results (33.1).

A similar study performed by Wang et al. in Taiwan reported a significant psychological impact on women diagnosed with abnormal Pap results, CIN, positive high-risk HPV test, and GW, compared with women with normal Pap test results. Female patients diagnosed with GW showed the highest psychological impact scores [47].

### Limitations

Only physicians treating a higher number of GW patients were included in the study, which may have led to an overestimation of GW when the resulting data was projected to the national level. GW patients who did not seek treatment were not included in the study, which may have underestimated the true prevalence in the Philippines, since weighting was applied. Moreover, overestimation of prevalence may have occurred as a high percentage of GW patients treated by IDSs were referred by other physicians. Despite the specific question about referrals and the exclusion of referred patients, some patients initially diagnosed by other specialists and then referred to IDSs for management may have been double-counted.

Potential bias related to the study information source may exist, as the recall of healthcare resource utilization estimated by physicians may be difficult to control. Additionally, the current study cannot determine whether the psychosocial impact on study participants drawn from the 4 Philippine cities is generalizable to the entire population.

## Conclusions

With an age, gender, and specialty weighted overall prevalence of 2.66%, GW poses a significant healthcare and economic concern in the Philippines. Median costs are estimated at 7121 PHP for men, and 7000 PHP for women (~$134–$132 USD), which is about 8.58% of the country’s average GDP per capita over the past two decades. In terms of HRQL, the study results suggest that a GW diagnosis in men and women has a negative impact on patient well-being and HRQL. For women, a greater impact was observed among GW patients compared with those with other selected HPV-related diseases. The baseline data in this study offers a foundation for measuring GW prevalence and its psychosocial impact on the overall population. Despite its limitations, this study provides an estimation of GW-related data in the Philippines not previously available.

## Data Availability

The data analyzed during the current study are available from the corresponding author on reasonable request.

## Appendix

**Table 7:** Participating Physicians by Region

q	PCP (*n* = 28)	DERM (*n* = 29)	OB/GYN (*n* = 43)	URO (*n* = 29)	IDS (*n* = 28)	Overall (*n* = 157)
Region						
Manila	22 (91.7%)	17 (68.0%)	23 (54.8%)	9 (42.9%)	15 (83.3%)	86 (66.2%)
Luzon	2 (8.3%)	3 (12.0%)	11 (26.2%)	4 (19.0%)		20 (15.4%)
Mindanao		5 (20.0%)	4 (9.5%)	5 (23.8%)	2 (11.1%)	16 (12.3%)
Visayas			4 (9.5%)	3 (14.3%)	1 (5.6%)	8 (6.2%)
Valid n	24	25	42	21	18	130

Percentages calculated over the corresponding valid n

*DERM* dermatologist, *IDS* infectious disease specialist, *OB/GYN* obstetrician/ gynecologist, *PCP* primary care physician, *URO* urologist

**Table 8:** HIP Questionnaire Scores by HPV-related Disease for Female Patients

HPV Disease (*n* = 182)							
	Abnormal PAP (*n* = 46)	Abnormal Pap/HPV+ (*n* = 45)	Precancerous lesions (*n* = 44)	External GW (*n* = 47)	No HPV Disease (*n* = 43)	Overall	*P*-value*
HPV Impact Profile Total Score							
Mean	44.81	40.69^1^	50.52	55.84^1^	48.06	47.99	0.0040
SD	20.09	18.29	19.47	18.71	20.56	19.94	
95% CI	(38.8; 50.8)	(35.2; 46.2)	(44.6; 56.4)	(50.3; 61.4)	(41.7; 54.5)	(45.4; 50.6)	
Worries and Concerns							
Mean	43.77	41.71^1^	54.06	58.72^1^	49.11	49.47	0.0178
SD	27.37	27.08	25.75	25.24	30.74	27.75	
95% CI	(35.6; 51.9)	(33.6; 49.8)	(46.2; 61.9)	(51.2; 66.2)	(39.5; 58.7)	(45.8; 53.1)	
Emotional Impact							
Mean	45.17	35.16^1^	48.49	57.66^1^	45.62	46.52	0.0004
SD	24.14	24.22	24.40	21.24	24.71	24.64	
95% CI	(38.0; 52.3)	(27.9; 42.4)	(41.1; 55.9)	(51.4; 63.9)	(37.9; 53.3)	(43.3; 49.8)	
Sexual Impact							
Mean	47.33	43.30	51.59	53.78	51.58	49.47	0.1738
SD	23.47	20.54	22.43	22.77	19.77	22.03	
95% CI	(40.3; 54.4)	(37.0; 49.5)	(44.8; 58.4)	(46.9; 60.6)	(45.1; 58.1)	(46.5; 52.4)	
Self-image							
Mean	41.30	41.44	41.88	49.60	44.31	43.72	0.2115
SD	19.91	18.03	20.38	18.44	21.09	19.66	
95% CI	(35.4; 47.2)	(36.0; 46.9)	(35.7; 48.1)	(44.1; 55.1)	(37.7; 50.9)	(41.1; 46.3)	
Partner Issues and Transmission							
Mean	49.71^1^	47.67^2^	57.46	66.22^1,2^	54.91	55.22	0.0069
SD	26.37	24.77	26.33	23.12	27.37	26.19	
95% CI	(41.9; 57.5)	(40.1; 55.3)	(49.3; 65.7)	(59.3; 73.2)	(45.6; 64.2)	(51.7; 58.8)	
Interactions with Doctors							
Mean	41.52	31.04^1^	46.36^2^	52.07^1,2^	41.30	42.46	0.0004
SD	22.07	18.61	24.26	25.14	22.05	23.41	
95% CI	(35.0; 48.1)	(25.4; 36.6)	(39.0; 53.7)	(44.5; 59.6)	(34.3; 48.3)	(39.4; 45.6)	
Control/Life Impact							
Mean	48.26	47.11	51.74	52.16	54.88	50.77	0.2894
SD	19.02	16.58	17.56	22.09	16.88	18.64	
95% CI	(42.6; 53.9)	(42.1; 52.1)	(46.4; 57.1)	(45.7; 58.6)	(49.6; 60.1)	(48.3; 53.2)	
HPV Impact Profile Total Score categorized							
No or little impact	20 (43.5%)	22 (48.9%)	12 (27.3%)	10 (21.3%)	15 (34.9%)	79 (35.1%)	
Moderate impact	20 (43.5%)	21 (46.7%)	24 (54.5%)	23 (48.9%)	21 (48.8%)	109 (48.4%)	
Heavy psychological impact	6 (13.0%)	2 (4.4%)	8 (18.2%)	14 (29.8%)	7 (16.3%)	37 (16.4%)	
Valid n	46	45	44	47	43	225	

*Analysis of Variance (ANOVA) test

Equal numbers show statistical differences between these means at level 0.05

*CI* confidence interval, *GW* genital warts, *HPV* human papillomavirus, *Pap* Papanicolaou test, *SD* standard deviation

## Abbreviations

CECA: Cuestionario Especifico para Condiloma Acuminado
CI: confidence interval
CIN: cervical intraepithelial neosplasia
DERM: Dermatologist
EQ-5D: EuroQol-5 Dimension
ER: emergency room
FM: family medicine
GP: general practitioner
GW: genital warts
HIP: HPV impact profile
HPV: human papillomavirus
HRQL: health related quality of life
IDS: infectious disease specialist
IMS: Intercontinental Marketing Services
OB/GYN: obstetrician/gynecologist
Pap: Papanicolaou
PCP: primary care physician
PHP: Philippine peso
SD: standard deviation
STI: sexually transmitted infection
TPP: third party payer
URO: urologist
VAS: Visual Analogue Scale
